# Network methods for diagonal integration of unpaired single-cell multiomics data: a review

**DOI:** 10.1093/bioinformatics/btag353

**Published:** 2026-06-02

**Authors:** Marcello Barylli, Joyaditya Saha, Tineke E Buffart, Jan Koster, Kristiaan J Lenos, Louis Vermeulen, Roland V Bumbuc, Vivek M Sheraton

**Affiliations:** Computational Science Lab, Informatics Institute, University of Amsterdam, Amsterdam, 1098 XH, The Netherlands; Laboratory for Experimental Oncology and Radiobiology (LEXOR), Cancer Center Amsterdam (CCA), Amsterdam University Medical Centers (location VUmc), Amsterdam, 1105 AZ, The Netherlands; Amsterdam Gastroenterology Endocrinology and Metabolism (AGEM), Amsterdam University Medical Centers (Location VUmc), Amsterdam, 1081 HV, The Netherlands; Oncode Institute, Utrecht, 3521 AL, The Netherlands; Laboratory for Experimental Oncology and Radiobiology (LEXOR), Cancer Center Amsterdam (CCA), Amsterdam University Medical Centers (location VUmc), Amsterdam, 1105 AZ, The Netherlands; Laboratory for Experimental Oncology and Radiobiology (LEXOR), Cancer Center Amsterdam (CCA), Amsterdam University Medical Centers (location VUmc), Amsterdam, 1105 AZ, The Netherlands; Laboratory for Experimental Oncology and Radiobiology (LEXOR), Cancer Center Amsterdam (CCA), Amsterdam University Medical Centers (location VUmc), Amsterdam, 1105 AZ, The Netherlands; Oncode Institute, Utrecht, 3521 AL, The Netherlands; Laboratory for Experimental Oncology and Radiobiology (LEXOR), Cancer Center Amsterdam (CCA), Amsterdam University Medical Centers (location VUmc), Amsterdam, 1105 AZ, The Netherlands; Amsterdam Gastroenterology Endocrinology and Metabolism (AGEM), Amsterdam University Medical Centers (Location VUmc), Amsterdam, 1081 HV, The Netherlands; Oncode Institute, Utrecht, 3521 AL, The Netherlands; Computational Science Lab, Informatics Institute, University of Amsterdam, Amsterdam, 1098 XH, The Netherlands; Department of Plastic, Reconstructive and Hand Surgery, Amsterdam Movement Sciences (AMS) Institute, Amsterdam UMC, Location VUmc, Amsterdam, 1081 HV, The Netherlands; Department of Molecular Cell Biology and Immunology, Amsterdam Infection and Immunity (AII) Institute, Amsterdam UMC, Location VUmc, Amsterdam, 1081 HV, The Netherlands; Computational Science Lab, Informatics Institute, University of Amsterdam, Amsterdam, 1098 XH, The Netherlands

## Abstract

**Motivation:**

Advances in single-cell sequencing have enabled multiomics profiling at unprecedented resolution; however, mass spectrometry-based single-cell proteomics (scMS) remains inherently destructive, precluding simultaneous transcriptomic capture. Unlike antibody-based methods such as CITE-seq, which permit paired profiling but are restricted to targeted protein panels, scMS provides unbiased, genome-scale coverage of the intracellular proteome yet necessitates post hoc integration of unpaired datasets. This diagonal integration challenge, where transcriptomes and proteomes are measured in separate cells lacking shared anchors, remains underserved by existing reviews, which focus predominantly on vertical integration strategies enabled by non-destructive assays.

**Results:**

We survey the complete computational pipeline for constructing mechanistic proteogenomic networks from unpaired single-cell data, covering: (i) unimodal network inference such as knowledge-based approaches, probabilistic graphical models, temporal directionality inference, and generative and foundation model strategies that establish the transcriptomic scaffold; (ii) cross-modal integration architectures such as network propagation, graph neural networks (scMRDR, scmFormer, scCotag), and consensus frameworks designed explicitly for the unpaired proteomics setting; and (iii) benchmarking paradigms spanning network reconstruction (BEELINE, GRETA, CausalBench) and multi-task integration evaluation (scMultiBench, SCMMIB), with guidance on metric selection under network sparsity and class imbalance. We identify three principal axes of future development: generative proteomic translation from transcriptomic precursors, inductive prior embedding in next-generation architectures, and perturbation-based causal benchmarking.

**Availability and implementation:**

This is a review article; no novel software is distributed. A curated benchmark resource table, methods starter guide, and per-method bottleneck annotations are provided in the Supplementary Material.

## 1 Introduction

Despite the progress made in the field of single cell -omics, an obvious remaining limitation is the fact that most single cell sequencing techniques only take into account transcriptomic changes between cells. Often, there appears to be a discrepancy between different biological layers concerning the same gene as there are numerous regulatory mechanisms at play. Therefore, to get a more comprehensive understanding, one must look to the emerging field of multiomics, where the relationships between different molecular levels are taken into account to explain phenotypic features. Through an efficient integration of different biological modalities, network based approaches have the potential to provide increased resolution at which biological information is obtained and interpreted. Network-based approaches allow modelling of the effect of perturbations spanning different biological layers instead of being limited to one (Kamimoto *et al.* 2023). Furthermore, such approaches allow us to decipher novel cellular populations that might not be uncovered through the analysis of one biological layer alone, as dynamic changes across layers are not sufficiently captured in a single-omics layer (Kuppe *et al.* 2022).

Combining single cell sequencing and multiomics network inference, however, remains a challenge. A distinction must be made regarding the nature of “proteomics” in current multiomics research. Single-cell mass spectrometry-based proteomics (scMS) offers unbiased, genome-scale coverage of the intracellular proteome (1000–5000+ proteins) (Leiserson *et al.* 2015), but is fundamentally destructive. This necessitates the integration of unpaired data: transcriptomes and proteomes measured in separate cells. This review focuses on the computational methods required to construct mechanistic proteogenomic networks from unpaired single-cell data, using the challenge of diagonal integration of scRNA-seq and scMS as a motivation. We survey each stage of this pipeline, from unimodal network inference to cross-modal alignment, to bridge the gap between mRNA potential and protein execution.

### 1.1 Network terminology

**Figure 1:**
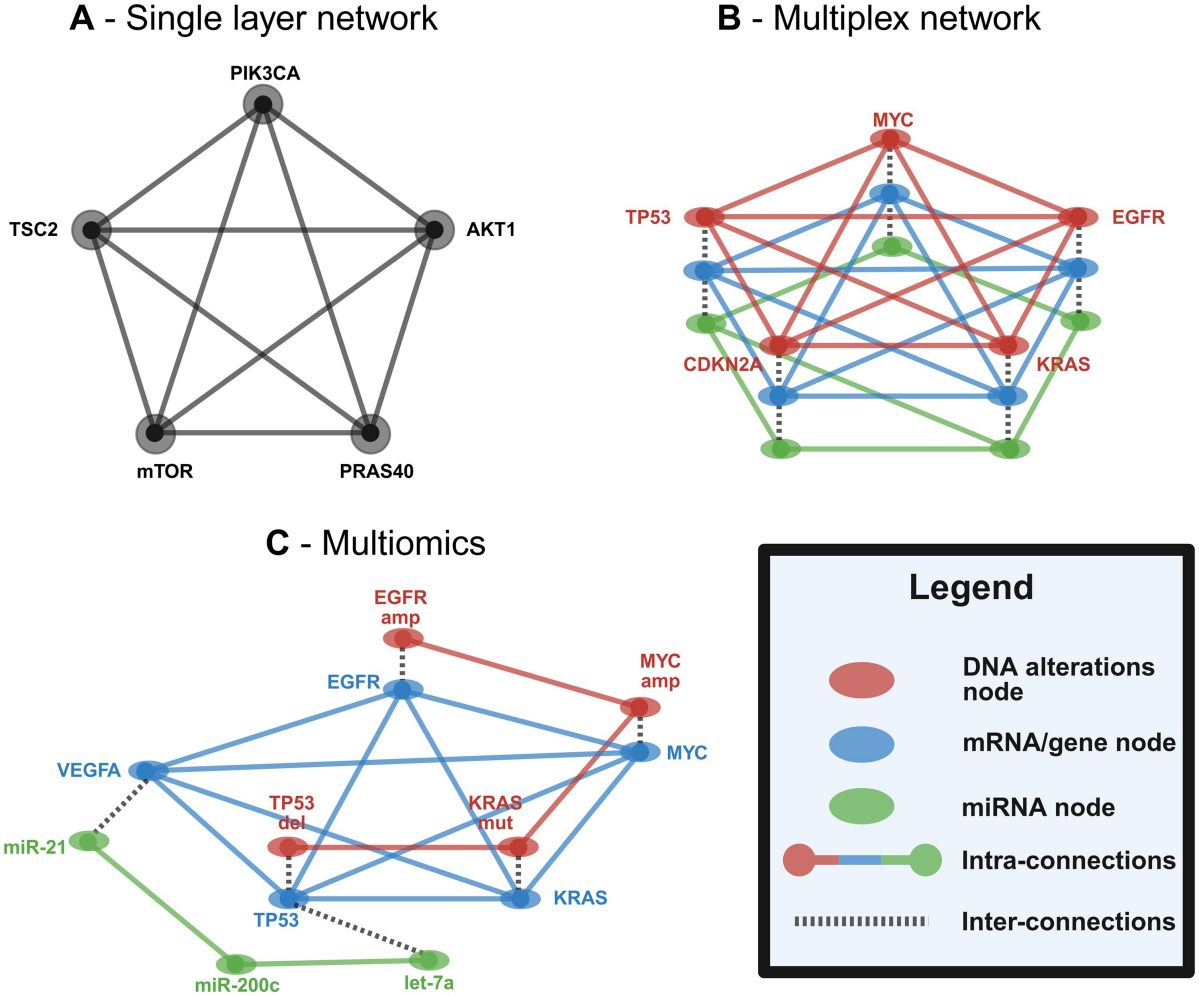
Network architectures for cancer multiomics integration. (A) A monoplex network showing the PI3K-AKT-mTOR signaling pathway as a single-layer protein-protein interaction network with one node type and one edge type (Kar *et al*. 2009). (B) A multiplex network where identical genes (TP53, MYC, EGFR, KRAS, CDKN2A) form nodes across multiple layers, each representing different relationship types (co-expression, transcription factor co-targeting, and microRNA co-targeting networks), connected by inter-layer edges (Cantini *et al*. 2015). (C) A multilayer multiomics network integrating different molecular scales such as DNA alterations (copy number variations and mutations), mRNA expression, and microRNA regulation, where nodes and edge types differ across layers, and cross-layer edges represent biological relationships such as gene dosage effects and post-transcriptional regulation (Kuijpers *et al*. 2021).

A graph G=(V,E) represents biological entities as nodes *V* and their interactions as edges *E* (Barabási and Pósfai 2016), commonly represented via an adjacency matrix *A*. Edges may be undirected or directed, the latter usually implying causal relationships (Fig. 1A).

Multiplex networks, as seen in Fig. 1B, model a single set of nodes from multiple *aspects* that characterize the nodes in different ways. Each aspect contains a separate edge set. The most flexible model is the heterogeneous multiomics network, depicted in Fig. 1C. These networks may contain multiple, potentially overlapping node sets and edge sets, with cross-layer edges possible between any two nodes of differing layers. Proteomics is intentionally excluded from the illustration in Fig. 1, as protein-level data does not share a common node identifier with the genomic layers depicted, mRNA—protein correlations are confounded by post-translational regulation, and the higher missingness characteristic of mass spectrometry-based proteomics precludes stable cross-layer edge definition in a multiplex framework, whereas proteomic integration is discussed separately.

### 1.2 Building networks from single cell data

Network inference from single cell data begins with a matrix containing cells as columns and genes as rows. Each cell represents a point in high-dimensional gene-space. Techniques such as t-SNE, UMAP, or PCA are commonly used to reduce the dimensionality upon which cells can cluster (Hinton and Roweis 2002, Jolliffe and Cadima 2016, McInnes *et al.* 2020). Gene-gene interactions are typically inferred on the basis of the correlation of their expression values across cells of a given type. Gene pairs exceeding a predefined similarity threshold form network links. This notion is termed “guilt by association” (Cowen *et al.* 2017). Although this omits causal relationships, it serves as a basis for downstream directionality inference. This results in a cell type-specific gene network, where each cell type is defined by its internal gene network. To link gene networks, cell-cell communication information is incorporated using tools such as CellPhoneDB (Efremova *et al.* 2020) or CellChat (Jin *et al.* 2021), which leverage ligand-receptor interactions shaping cellular communities. This creates a multi-scale “network of networks” between different cells (Hammoud and Kramer 2020). An illustration of such a multi-scale network is provided in the Supplementary Material, available as supplementary data at *Bioinformatics* online.

### 1.3 Classifying single-cell multiomics integration strategies

The construction of high-resolution network models requires the computational alignment of various -omics levels. To clarify the landscape of integration strategies, we adopt a taxonomy based on two orthogonal axes: the architectural stage of integration and the data relationship between modalities (Adossa *et al.* 2021, Rong *et al.* 2024). A detailed discussion on the architectural strategies and data relationships is available in the Supplementary Material, available as supplementary data at *Bioinformatics* online.

#### 1.3.1 The diagonal proteomics gap

While several recent reviews have addressed multiomics integration, they predominantly focus on vertical integration scenarios enabled by non-destructive assays. Reviews covering the integration of CITE-seq and scATAC-seq (Stoeckius *et al.* 2017, Stuart *et al.* 2019) leverage the abundance of paired data to discuss methods such as Seurat WNN and MOFA+. However, these reviews often overlook the unique challenges of mass spectrometry-based proteomics (scMS). Unlike CITE-seq, which targets surface markers using antibodies, scMS provides an unbiased view of the intracellular proteome but is destructive, rendering vertical integration impossible for most workflows.

#### 1.3.2 The diagonal proteomics gap and post-hoc integration

While several recent reviews have addressed multiomics integration, they predominantly focus on vertical integration scenarios enabled by non-destructive assays (e.g. CITE-seq, scATAC-seq) (Stoeckius *et al.* 2017, Stuart *et al.* 2019), or on the scRNA/scATAC axis where “gene activity scores” serve as biological priors for feature matching (Cao and Gao 2022). These approaches overlook the unique challenges of mass spectrometry-based proteomics, which requires distinct diagonal integration strategies [e.g. single-cell Multiomics Regularised Disentangled Representations (scMRDR) (Thompson *et al.* 2024), scCotag (Li *et al.* 2025), scmFormer (Xu *et al.* 2024)] to bridge the translation gap without shared cell anchors. Such diagonal integration relies on *intermediate integration architectures* capable of statistical translation, leveraging prior biological knowledge or graph-guided priors [e.g. GLUE (Cao and Gao 2022), scMoGNN (Wen *et al.* 2022)] to align manifolds in a shared latent space. Emerging approaches in this domain also include causal modelling frameworks such as HALO (Mao *et al.* 2025), which attempt to distinguish coupled from decoupled variations in unmatched data.

Furthermore, reviews covering diagonal integration often focus on the scRNA/scATAC axis, where “gene activity scores” serve as biological priors for feature matching (Cao and Gao 2022). Our review focusses on the diagonal integration of scRNA-seq and scMS, a domain that requires distinct computational strategies [e.g. scMRDR (Thompson *et al.* 2024), scCotag (Li *et al.* 2025), scmFormer (Xu *et al.* 2024)] to bridge the translation gap without shared cell anchors. This distinction is critical for inferring mechanistic networks that reflect functional protein execution rather than just transcriptional potential.

#### 1.3.3 Post-hoc diagonal integration

Diagonal integration requires *intermediate integration architectures* capable of statistical translation. This means leveraging prior biological knowledge or graph-guided priors [e.g. GLUE (Cao and Gao 2022), scMoGNN (Wen *et al.* 2022)] to align manifolds in a shared latent space. We therefore focus on diagonal integration methods in the subsequent sections, as they represent the critical post-hoc solution. Emerging approaches in this domain also include causal modelling frameworks such as HALO (Mao *et al.* 2025), which attempt to distinguish coupled from decoupled variations in unmatched data.

### 1.4 Connecting layers via joint embedding

The central operation underlying these architectures is joint embedding (or reduction in joint dimensionality) (Lee *et al.* 2020). This transforms heterogeneous data types, which share no common features or cells, into a joint, interpretable latent vector space. joint embedding allows for the inference of correspondence via manifold alignment (Hetzel *et al.* 2021). This effectively solves the “statistical translation” problem inherent to post-hoc analysis. When applied in a network context, these embeddings serve as the foundation for matrix factorization (see Supplementary Material, available as supplementary data at *Bioinformatics* online), network propagation, and Graph Neural Network (GNN) architectures described in Section Network Integration and Analysis.

## 2 Network inference and integration methods

The two most relevant method classes in the context of network-based single cell multiomics are as follows:

- Methods that infer network structure directly: useful for constructing networks from single cell expression data corresponding to one of the different biological levels.
- Methods that connect levels: infer missing intra- and cross-level links. This type of task can be construed as integrating the multiomics network.

The split between the single cell and multiomics methods is summarized in the summary of methods (Table 1, available as supplementary data at *Bioinformatics* online).

### 2.1 Network inference

#### 2.1.1 Knowledge-based network inference

When determining an optimal path towards the solution of a problem, the use of prior knowledge serves as a good starting point. Knowledge graphs (KG) serve as structured graphical representations of heterogeneous graphs, modelling specific relationships between entities. For example, they can describe the empirically obtained regulatory relationships between genes in gene regulatory networks (GRNs). A fundamental example of KG is Gene Ontology (GO) (Aleksander *et al.* 2023). The Precision Medicine Knowledge Graph (PrimeKG), which integrates 20 modalities not limited to molecules, is a versatile example of a KG platform based on ontology (Chandak *et al.* 2023). This knowledge graph, which consists of approximately 129 000 nodes and over 4 000 000 edges, allows the inference of associations between various diseases and drugs.

#### 2.1.2 Probabilistic graphical models

In contrast to knowledge-driven graph generation, a fundamentally data-driven approach of graph generation comes in the form of relevance networks (Werhli *et al.* 2006, Ye and Guo 2022). Such correlation-based methods infer interactions from statistical relationships between entities. In the case of transcriptomics data, co-expression of genes could act as correlation measures. Most commonly, Pearson correlation or regression is used to determine the proximity of pairs of genes in cell-space.

If the variables are assumed to be normally distributed, we arrive at a form of PGM known as Gaussian Graphical Models (GGM). Here, connections between nodes are simply inferred via an inverse covariance matrix $\Sigma^{-1}$. Recent extensions of GGMs, such as Augmented High-Dimensional Graphical Lasso (AhGlasso), have been developed to incorporate prior knowledge (knowledge graphs) with edge weights for improved global network learning in high-dimensional settings (Zhuang *et al.* 2021). The Gaussianity constraint is relaxed in Mixed Graphical Models (MGM) (Lauritzen 1996). The popularity of these methods stems from their straightforward interpretation via conditional dependencies, allowing the distinction between direct and indirect effects, as well as their computational efficiency (Altenbuchinger *et al.* 2020).

#### 2.1.3 Temporal data-based edge directionality inference

PGMs are straightforward and comparatively efficient in terms of computation. However, inferring directionality can only be done with time-stamped data or prior knowledge. If such information is available, it can be leveraged by causal models. Networks constructed using causal models have the benefit of allowing for directed relationships between nodes (Hetzel *et al.* 2021), as well as the detection of subnetworks (Agamah *et al.* 2022). For example, models such as SINCERITIES (Papili Gao *et al.* 2018), make use of time-stamped omics datasets. In this approach, regression analysis is used which is a statistical method to estimate the dependency relationships among variables.

#### 2.1.4 Knowledge-based edge directionality inference

In the absence of temporal information, prior knowledge could alternatively be used to establish directionality between nodes. One such correlation-based method focused on single cell transcriptomics was developed by Iacono *et al.* (2019). This method uses a specialized form of correlation based on transformed variables (Z-scores). A knowledge graph approach informs directionality using gene ontology, providing a subset of “regulators of gene expression.” Not to be confused with the conditional dependence of GGMs, the conditional probability *P* (Y—X) of two genes X and Y corresponds to a directionality of X → Y. This type of directed relationship expresses the probability that gene Y is being expressed, given the knowledge that X is being expressed. Directed edges can then be established between a set of multiple genes, resulting in a Bayesian network, or directed acyclic graph (DAG) (Saint-Antoine and Singh 2020).

A major drawback of these Bayesian methods is their computational cost, which limits them to the analysis of small datasets. Furthermore, cyclical relationships in the form of feedback loops cannot be captured with most methods, since the framework utilizes DAGs (Ye and Guo 2022). This limitation detracts from the applicability of GRNs, which are characterized by such cyclical motifs.

#### 2.1.5 Boolean control networks

Boolean control networks (BCNs) (Schwab *et al.* 2020) are a class of models that address the issue of scaling, while at the same time offering unique benefits over correlation models in terms of executability. In BCNs, gene activation states are binarized from expression signatures, upon which logical update functions are found to inform gene relationships (Ye and Guo 2022). The simplifying nature of binarizing node states facilitates efficient scaling of network size (Gyori *et al.* 2017). Recently, the inference of Boolean networks has been simplified, making the process less computationally expensive (Parul Maheshwari *et al.* 2022). Ye and Guo (2022) developed a Prediction Logic Boolean Implication Network (PLBIN), which improves the basic models by adding implication relationships between variables via logical rules. Although Boolean networks offer benefits in terms of executability, binarizing gene expression leads to loss of information, since real expression values are continuous.

#### 2.1.6 Generative and foundation model inference

A recent paradigm shift is the transition from static correlation-based inference to generative modelling. Unlike PGMs, which define networks via conditional dependence, generative models reconstruct GRNs by learning the data generation process itself.

**Diffusion models:** Diffusion Probabilistic Models (DPMs), originally applied to image generation, have been adapted for GRN inference. The core premise is that the observed single-cell expression data represents a diffused state, and the underlying regulatory network can be recovered by learning the reverse denoising process. DigNet (Wang and Liu 2025) frames GRN inference as a discrete diffusion generation problem. Starting from a random graph, DigNet iteratively refines the adjacency matrix, recovering edges conditioned on the expression data. This allows for the generation of cell-specific networks and is robust to the sparsity inherent in scRNA-seq. Continuing this progress, Planet (Xu *et al.* 2025b) incorporates an attention-guided mechanism. It uses a “Triple Hybrid-Attention Transformer” to capture long-range dependencies and ensure global structural consistency during the diffusion process.

**Foundation models:** Large-scale foundation models (scFMs) pre-trained on millions of single cells are now being hybridized with network approaches. scRegNet (Kommu *et al.* 2025) addresses the topological limitations of early transformers by fusing semantic embeddings from pre-trained scFMs (like scBERT or scFoundation) with structural embeddings from GNNs. This hybrid approach leverages both the data-driven syntax learnt from millions of transcriptomes and the explicit biological priors of regulatory graphs. Similarly, Cell-GraphCompass (Fang *et al.* 2025) represents a “cells-as-graphs” foundation model, pre-trained on 50 million cells where topological structure is embedded directly into the model architecture, rather than treating genes as a linear sequence. For scalability, scMamba (Yuan *et al.* 2025) introduces State Space Models (SSMs) to single-cell genomics, allowing linear scaling to model the context of the whole-genome without the need for aggressive feature selection.

### 2.2 Network integration and analysis

The network inference methods described in the preceding section operate within a single modality, typically scRNA-seq. These unimodal networks serve as the basis upon which cross-modal integration is built. In diagonal integration, where no shared cells exist between modalities, the topology of a transcriptomic network provides the prior structure needed to anchor proteomic measurements to their regulatory context. For instance, gene, gene co-expression relationships inferred from scRNA-seq define the manifold geometry that alignment methods such as GLUE (Cao and Gao 2022) and scConfluence (Samaran *et al.* 2024) exploit to project unpaired proteomic profiles into a shared latent space. The fidelity of this scaffold directly constrains the quality of the resulting multiomics network: errors in unimodal inference, whether from dropout noise or spurious correlations, propagate into the integrated representation, producing false cross-layer edges or obscuring true regulatory links. Recent work on bridge integration, such as scPairing (Niu *et al.* 2025), further demonstrates that the quality of unimodal embeddings is the rate-limiting factor for generating realistic paired multiomics profiles from unpaired data. The methods that follow therefore either operate directly on the inferred unimodal structures defined in the preceding section, or learn joint representations from the raw data itself; in both cases, the principles and biological priors outlined above inform the architectural choices that guide cross-modal alignment.

#### 2.2.1 Network propagation

**Figure 2:**
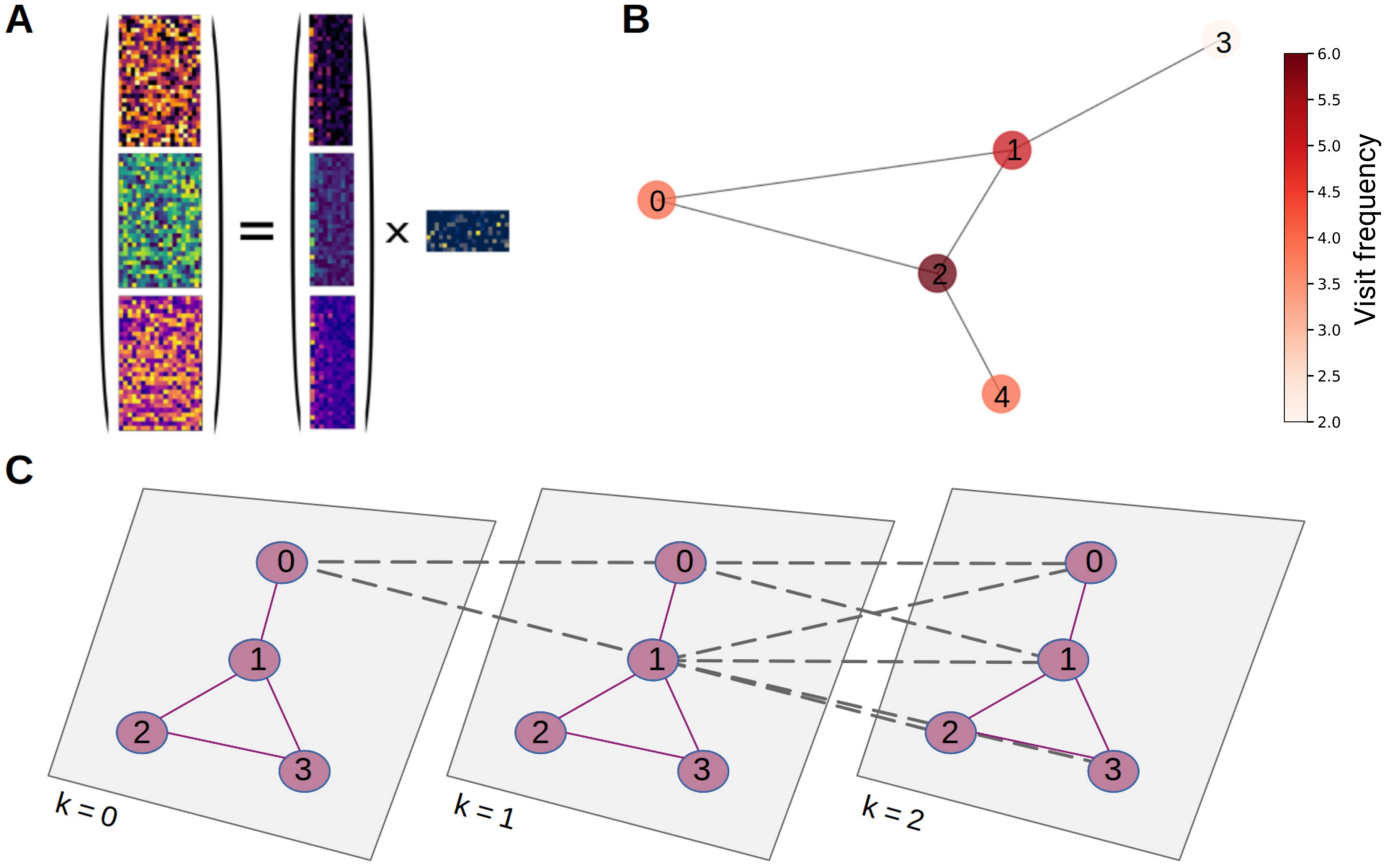
Comparison of integration methods. (A) The decomposition of three data matrices from different sources into lower dimensional matrices via factors. (B) Topological node information obtained via random walks, where color intensity indicates visit frequency. (C) Three layers of a graph neural network, indicated by *k*. Dashed lines indicate message passing, where information is aggregated at node 0.

An alternative class of methods, summarized as network propagation, can address the challenge of capturing non-linear relationships. Designed as a powerful and flexible framework that has found use in all disciplines in various forms, including PageRank and heat diffusion (HD), network propagation is a dominant technique in systems biology (Hetzel *et al.* 2021). An intuitive instance of this framework is the random walk (RW). Here, a “walker” moves from node to node in the network, its path being indicative of the nodes’ connectivity and position in the network. Figure 2B depicts the averaged results of a random walk starting at such a source node (Node 0). Highly connected nodes will be visited frequently, indicating their status as hubs. Random walk with restart (RWR) is a variation on this approach which adds a fixed probability of returning to the source node at each step, thereby confining diffusion to local neighbourhoods even at steady state.

Cowen *et al.* (2017) elucidate the efficacy of this method in detecting cancer driver genes, whose somatic mutations contribute to tumor development. The underlining of this approach is the notion that genes related to a disease are more likely to interact with each other than with randomly selected genes. Therefore, biological prior knowledge, such as known cancer driver genes, is superimposed on the network. Using these nodes as sources, the information is propagated across the network, amplifying the signal and revealing clusters of highly significant nodes.

#### 2.2.2 Graph neural networks and hypergraphs

GNNs and their sub-variants, graph convolutional nets (GCN) and graph attention nets (GAT), form a powerful class of methods for capturing complex interactions and nonlinearities in biological data (Zhang *et al.* 2021) (see Fig. 2C for a schematic depiction of spatial graph convolution, or message passing). GNNs are flexible tools that have proven to be capable of handling large-scale, heterogeneous, and complex networks. They are able to extract the topology and key features of the data on a massive scale (Hu *et al.* 2020).

Recent advances have expanded the GNN framework to address the specific topological complexities of multiomics data. scHyper (Li *et al.* 2024) uses hypergraph neural networks to model these high-order correlations. By constructing hyperedges that can connect an arbitrary number of nodes (e.g. a set of co-accessible chromatin peaks and their target genes), scHyper achieves higher performance in atlas-scale integration tasks compared to traditional pairwise GNNs.Single-cell Multiomics Graph Attention Network (Liao *et al.* 2025) introduces a multi-head attention mechanism that dynamically weights the importance of neighbors. This allows the model to respond to informative biological signals while suppressing technical noise and dropout events, improving the annotation of cell-types in heterogeneous tissues.

These newer models are based on established frameworks such as DeepMAPS (Ma *et al.* 2023), which uses heterogeneous graph transformers (HGT) to explicitly model different edge types (e.g. cell-gene versus gene-gene). By assigning relation-specific parameters, DeepMAPS differentiates the semantic meaning of regulatory links from co-expression links. More recent work highlights the strengths of HGTs in modelling disparate modalities (e.g. chromatin peaks versus gene expression) (Wang *et al.* 2024). In these settings, HGTs prevent the loss of subtle signals that occurs during the transmission of standard messages by explicitly modelling the structural information carried by different types of nodes (Yang *et al.* 2025).

More broadly, new GNN architectures are emerging to tackle the diagonal integration of unpaired mass spectrometry data. scMRDR (Sun *et al.* 2026) uses a disentangled variational autoencoder (VAE) to separate modality-specific noise (such as MS missingness) from shared biological signals. This allows for the integration of unpaired scRNA-seq and scMS data by aligning their latent manifolds, predicting protein abundance from transcriptomic profiles despite the lack of shared cells. Similarly, scmFormer (Xu *et al.* 2024) adapts Transformer architectures to perform multi-task learning, treating the translation between transcriptomic and proteomic modalities as a sequence-to-sequence problem, enabling large-scale integration of unpaired datasets. Furthermore, scCotag (Li *et al.* 2025) leverages Co-Optimal Transport to learn mappings between unpaired datasets without assuming that all cells are alignable, addressing the unbalanced nature of scMS data. ScTGCN (Kan *et al.* 2025) leverages Graph Convolutional Networks (GCN) to transfer labels and embeddings from source scRNA-seq data to target scMS data, explicitly modelling topological conservation between the modalities.

Addressing the trade-off between information propagation and distinctiveness of features requires topological considerations. Although data-driven k-Nearest-Neighbor (kNN) graphs effectively capture abundant cell states, they risk amplifying noise in sparse datasets or fragmenting continuous trajectories (Cao *et al.* 2024). In contrast, incorporating biological priors, such as Protein-Protein Interaction (PPI) or Ligand-Receptor (LR) networks, can bridge technical dropouts and guide inference in spatially restricted data (Mao *et al.* 2023, Cao *et al.* 2024). To mitigate negative transfer from irrelevant priors, recent methods use dual-channel architectures or adaptive graph rewiring, allowing the model to dynamically weigh observed data against prior knowledge (Zheng *et al.* 2024, Gao *et al.* 2025). To further overcome data scarcity and technical noise in these network topologies, contrastive learning frameworks maximize the agreement between augmented data views, providing a self-supervised signal that enhances network robustness against dropout and batch effects without requiring paired labels (Lin and Ou-Yang 2023, Guan *et al.* 2026). Meanwhile, when prior regulatory knowledge is extremely scarce, graph meta-learning approaches [such as Meta-TGLink (Yu *et al.* 2025)] use bi-level optimization across diverse source datasets to enable network inference in a few-shots in unseen cell types. Finally, transfer learning intrinsically links these graph models to larger reference atlases, allowing structural interaction rules learnt on massive atlas-scale data to be seamlessly transferred and fine-tuned for specialized, smaller-scale multiomics integration tasks (Xu *et al.* 2025a, Yuan and Duren 2025).

#### 2.2.3 Consensus and post-hoc integration

Given the stochastic nature of single-cell data and the sensitivity of inference algorithms to parameter choices, relying on a single inferred network is increasingly seen as inadequate. Post-hoc integration, i.e. the refinement and combination of networks *after* initial inference, has emerged as a standard requirement for robust analysis.

Consensus frameworks aim to mitigate the bias of individual mathematical formalisms. Consensus algorithm FOr scRNA-seq data (Lodi *et al.* 2024) uses a Borda count ranked-choice voting system to aggregate networks inferred by multiple independent algorithms. Furthermore, post-hoc integration is increasingly used to bridge distinct biological layers, such as transcription Regulation (GRNs) and signaling (PPIs). Shusi (Zhang *et al.* 2025) uses Large Language Models (LLMs) to predict context-specific protein interactions, which are then integrated with inferred GRNs to map the complete path from receptor signaling to nuclear transcription. Additionally, scConfluence (Samaran *et al.* 2024) uses Unbalanced Optimal Transport to integrate datasets with discrepant cell populations, to merge high-throughput transcriptomics with lower-throughput targeted proteomics.

## 3 Method validation

Although all of the methods listed above will provide some output, it is essential to ensure that these results align with biological reality to a sufficient degree, both in inference and integration settings.

### 3.1 Traditional network inference benchmarking

One way to evaluate performance is via comparison to ground truth networks. Chen *et al.* used synthetic networks generated with GeneWeaver (Baker *et al.* 2012) and gene relation networks from publicly available experimental data. The corresponding datasets describing both types of networks were generated from the network structures. This allowed the true network structure to be known and provided input data for the models to be tested. Model performance was evaluated using Receiver Operating Characteristic (ROC) and Precision-Recall (PR) curves. The results showed that performance on the experimental data was poor across the board, while single cell-specific methods performed marginally better on one of the simulated datasets.

Pratapa *et al.* used a similar approach for benchmarking (Pratapa *et al.* 2020) through comparison with ground truth networks taken from synthetic networks, literature-curated Boolean models and diverse transcriptional regulatory networks. The authors developed BEELINE, a comprehensive evaluation framework to assess GRN reconstruction performance for single cell expression data. The framework allows for comparison of the accuracy, stability, and efficiency of the methods across single cell datasets. By providing a uniform pipeline that includes pre-processing, parameter estimation, and post-processing, comparability is ensured. The main performance metric is the Area Under Precision-Recall Curve (AUPRC) ratio, which is the AUPRC of the method to be tested divided by that of a random predictor.

However, more recent benchmarking efforts have highlighted a critical “causality gap” in these evaluations. The GRETA framework (Gene Regulatory nETwork Analysis) (Badia I Mompel 2025) systematically evaluated state-of-the-art multimodal inference methods and found that while models often perform well on predictive metrics (reconstructing observed expression), they fail on mechanistic metrics (predicting the outcome of TF perturbations). This distinction is vital for ensuring that inferred networks represent true causal regulatory links rather than just co-expression states. To address this limitation, CausalBench (Chevalley *et al.* 2025) provides a large-scale benchmark using real-world single-cell perturbation data (CRISPR knock-outs, drug treatments) to evaluate whether inferred networks can predict the effects of interventions, a gold standard for causal inference. Methods are evaluated not only for their ability to reconstruct the static network topology but also for their capacity to predict changes in gene expression under perturbation, distinguishing true regulatory causality from spurious correlations.

### 3.2 Comprehensive multimodal integration benchmarking

The rise of diagonal and mosaic integration methods for unpaired multiomics data has necessitated new benchmarking frameworks that assess performance across multiple downstream tasks simultaneously. scMultiBench (Liu *et al.* 2025) provides a systematic multi-task evaluation framework that benchmarks 40 integration methods across four data integration categories (vertical, diagonal, mosaic, and cross) on 64 real datasets and 22 simulated datasets. Critically, scMultiBench evaluates methods on six distinct tasks: dimension reduction, batch correction, cell type classification and clustering, imputation, and feature selection. This multi-task approach reveals that no single method dominates in all tasks, for instance, StabMap excels at dimensionality reduction and clustering, while scMoMaT and Multigrate show superior batch correction performance (Liu *et al.* 2025). The framework also assesses computational scalability by benchmarking both memory usage and runtime on datasets ranging from thousands to millions of cells, providing practical guidance for atlas-scale analyses.

Similarly, Single-cell Multi-modal Integration Benchmark (SCMMIB) (Fu *et al.* 2025) evaluates 65 methods in 40 integration algorithms, explicitly distinguishing between paired integration, unpaired diagonal integration, and unpaired mosaic integration scenarios. SCMMIB emphasizes four key dimensions: usability (ease of installation and execution), accuracy (biological correctness of integrated representations), robustness (stability across data perturbations and hyperparameter choices) and scalability (computational efficiency on large datasets). Importantly, SCMMIB has been extended to evaluate LLM-driven analysis agents, reflecting the growing role of foundation models in single-cell analysis (Fu *et al.* 2025). The benchmark workflow is publicly available (see resources, Table 2, available as supplementary data at *Bioinformatics* online), enabling a continuous community-driven evaluation of emerging methods.

### 3.3 Transfer learning and reproducibility assessment

Beyond the accuracy of held-out test sets, the reproducibility and generalizability of inferred networks across independent datasets is a critical but often overlooked validation criterion. Kang *et al.* (2021) highlight the importance of this approach by going beyond simulated datasets for validation via a reproducibility study. The rationale in this study is that the inferred networks should strongly overlap with each other, given the shared biological ground truth. This principle has been formalized in recent benchmarks through transfer learning evaluations, in which models trained on one dataset are applied to independent cohorts of the same biological condition (Heumos *et al.* 2023, Jumde *et al.* 2024). Methods that learn generalizable regulatory principles should maintain consistent network topology and edge weights across batches, while overfit methods show substantial variability (Heumos *et al.* 2023). Transfer learning benchmarks also assess the generalization between species, e.g. whether a GRN inferred from mouse hematopoietic data can predict regulatory relationships in human hematopoiesis (Jumde *et al.* 2024). These cross-dataset and cross-species evaluations are particularly relevant for multiomics integration, where the relationship between modalities (e.g. mRNA-protein correlation) should be reproducible across studies if integration methods are capturing true biological coupling rather than batch-specific artefacts.

### 3.4 Distinguishing metrics: network reconstruction versus integration quality

Although AUROC and AUPRC are standard metrics for evaluating binary classification performance, their interpretation differs substantially between network reconstruction and integration tasks, and the appropriate selection of metrics depends on the class imbalance and cost asymmetry of the problem (Davis and Goadrich 2006, Saito and Rehmsmeier 2015). **For network reconstruction**, AUPRC is generally preferred over AUROC when evaluating GRN inference because regulatory networks are highly sparse. The BEELINE framework standardizes this by reporting AUPRC ratios (method AUPRC divided by random predictor AUPRC) to account for baseline performance under different sparsity levels (Pratapa *et al.* 2020).

**For integration quality assessment**, the evaluation paradigm shifts from edge-level classification to cell-level embedding quality. Here, AUROC is commonly used for query-to-reference mapping tasks, where the goal is to classify query cells into reference cell types based on integrated embeddings (Heumos *et al.* 2023). However, when cell type distributions are imbalanced (e.g. rare cell populations), AUPRC again becomes more informative to assess whether integration methods preserve rare cell type identities (Luecken *et al.* 2022). Critically, integration benchmarks also use network-independent metrics such as silhouette scores (measurement of cell type clusters), Local Inverse Simpson’s Index (LISI) for batch mixing (Korsunsky *et al.* 2019), and graph connectivity preservation (Luecken *et al.* 2022). Recent frameworks such as BioLLM explicitly evaluate *gene regulatory network fidelity* as an integration quality metric, assessing whether integrated embeddings preserve the underlying regulatory structure inferred from single-modality data (Qiu *et al.* 2025). This highlights a key distinction: network reconstruction methods are evaluated on their ability to predict edges from expression data, whereas integration methods are evaluated on whether they preserve known biological structure (including networks). AUPRC provides a more robust evaluation under sparsity (Saito and Rehmsmeier 2015, Luecken *et al.* 2022). For a comprehensive multiomics quick start guide (with datasets) and summary of bottlenecks associated with various methods, readers are advised to look at the Starter Guide and Bottlenecks sections in the Supplementary Material, available as supplementary data at *Bioinformatics* online.

## 4 Outlook: from descriptive maps to predictive engines

The field is rapidly advancing from static network reconstruction to the development of executable causal engines. The next frontier lies in three specific domains, 1. Bridging the Protein Gap via Generative Hallucination: As scMS remains destructive, diagonal integration will transition from manifold alignment to generative translation. Future models will likely utilize large-scale foundation models (scFMs) to “hallucinate” the intracellular proteome from transcriptomic precursors, treating the translation gap as a sequence-to-sequence problem rather than a statistical alignment task. 2. Inductive Prior Embedding: Rather than using Knowledge Graphs for post-hoc validation, the next generation of “Cells-as-Graphs” architectures must incorporate biological priors directly into the model’s inductive bias. Moving beyond standard transformers to State Space Models like scMamba will enable models to process whole-genome structural contexts without the quadratic scaling bottlenecks currently limiting long-range regulatory inference. 3. Causal Benchmarking: Validation must move beyond AUPRC/AUROC of static edges. The standard is shifting toward perturbation fidelity: the ability to predict the transcriptome-wide response to a specific TF knockout or drug treatment. Ultimately, the goal is to move past “guilt-by-association” toward models that can simulate *in silico* perturbations, providing a functional bridge between mRNA potential and protein execution.

## Supplementary Material

btag353_Supplementary_Data

## Data Availability

No new data were generated or analysed in support of this research.
