# Supplementary material for: Network methods for diagonal integration of unpaired single-cell multiomics data: a review
Source: Bioinformatics. 2026 Jun 2;42(6):btag353. doi: 10.1093/bioinformatics/btag353 (PMC13284991; doi:10.1093/bioinformatics/btag353)
Supplement: btag353_Supplementary_Data [file btag353_supplementary_data.pdf]

Bioinformatics, 2026, pp. 1–6
Supplementary Material

Supplementary Material: Network Methods for Diagonal Integration of Unpaired Single-Cell Multi-Omics Data

Architectural Strategies and Data Relationships

The integration of multi-omics data can be characterised along two orthogonal axes: the stage of the computational pipeline at which integration occurs, and the structural relationship between the datasets being combined. This section expands on the taxonomy introduced in the main text.

Architectural Strategies: Early, Intermediate, and Late

This axis categorises methods based on when integration occurs in the computational pipeline (Adossa et al., 2021):

Early Integration (Concatenation): Raw feature matrices from different modalities are concatenated into a single input matrix before dimensionality reduction. Although straightforward, it typically faces difficulties with the disparate statistical distributions of different omics layers (e.g. continuous fluorescence intensity in CITE-seq vs. sparse counts in scATAC-seq) (Adossa et al., 2021; Argelaguet et al., 2021).

Late Integration (Consensus): Each modality is analysed independently to derive layer-specific abstractions such as clusters, which are then fused. This often fails to capture synergistic interactions between modalities, such as the coupling between distal enhancer accessibility and gene expression (Adossa et al., 2021).

Intermediate Integration (Joint Embedding): This learns a joint latent representation (manifold) simultaneously from all input modalities. By mapping disparate feature spaces into a shared space, it enables the alignment of cellular states based on biological similarity rather than direct feature overlap (Adossa et al., 2021).

Data Relationships: Vertical, Horizontal, and Diagonal

The relationship between samples and features defines the specific integration challenge, often described in the "mosaic integration" framework (Argelaguet et al., 2021; Kriebel and Welch, 2022):

Vertical Integration (Matched): Multiple modalities are measured from the *same single cell* (e.g. 10x Multiome, CITE-seq). The cell correspondence is known *a priori*, acting as a hard anchor (Stoeckius et al., 2017).

Horizontal Integration (Batch Correction): Datasets of the *same modality* (e.g. scRNA-seq) are integrated across different batches. The features (genes) act as anchors to align the data (Stuart et al., 2019).

Diagonal Integration (Unmatched): Different modalities (e.g. scRNA-seq and scATAC-seq) are profiled in *different* sets of cells. This is the most challenging scenario, as the data matrix contains information only in diagonal blocks, with no shared cell or feature anchors (Argelaguet et al., 2021; Cao et al., 2021).

Matrix Factorization

Joint embedding is central to heterogeneous data integration. Genes may be described as points in cell-space, where the dimensionality of the space is determined by the number of measurements. The embedding operation enables genes to be cast into a shared space defined by measurements on multiple levels. For example, a gene's expression may be measured via scRNA-Seq as well as single cell proteomics, resulting in two separate data matrices. The integration of these disparate data types commonly involves dimensionality reduction, which simplifies the feature space by assuming that phenomena of interest are described by a core set of latent features. A key technique in this process is matrix factorization (MF), a broad category of methods that reduce a matrix into lower-dimensional matrices via linear transformations. MF describes a dataset as a product of two matrices: one that represents a shared latent space for the data points (e.g. cells) and another that represents the contribution of the original features (e.g. genes) to that latent space. Furthermore, non-negative matrix factorization (NMF), which imposes a positivity constraint on the factors, is widely used in biological network analysis for tasks like multiomics data integration or module detection (the latter of which is used to probe the modular structure of gene networks that supports biological functionality).

Recent research has employed matrix factorization in a multiomics context of a single cell where vertical integration is not possible due to batch discrepancies. Introduced as the "mosaic integration" problem by Argelaguet et al., this form of integration targets data from separate experiments performed at different biological levels. To address the challenges resulting from input matrices with discrepancies in dimensions, Kriebel et al. (2022) developed a non-negative matrix factorization method to integrate multiomics datasets. The key contribution of the method, termed UINMF, is the use of an unshared metagene matrix. This unshared matrix allows for the incorporation of features that are only present in a subset of data types, whereas previous methods could only use data that were shared by all modalities. UINMF allows the integration of single cell RNA-Seq, spatial transcriptomics, single-nucleus chromatin accessibility,

© The Author(s) 2026. Copyright and licence statements to be updated by the publisher during production.

and mRNA expression sequencing (SNARE-Seq) data (Chen et al., 2019) between species datasets.

Thus, non-negative matrix factorization methods are effective and straightforward, offering interpretability due to the additivity of its factor matrices (Lee and Seung, 1999). However, a major limitation of current matrix factorization methods is the assumption of linearity. The decomposition of the data matrices into their latent factors is linear, which means that the linear reconstruction of the said matrices cannot capture complex non-linear relationships across layers (Lee et al., 2020). Furthermore, exceedingly large matrices are prohibitive, limiting the use of this framework for genome-scale multiomics data at the single cell level.

Bottlenecks in Single-Cell Multi-Omics Integration

Beyond the algorithmic choices described above, several practical and computational challenges limit the applicability of current methods. We summarise the three principal bottlenecks below.

Data Preprocessing and Normalization

The diagonal integration of unpaired single-cell transcriptomics and proteomics data requires careful consideration of preprocessing strategies that accommodate the distinct technical characteristics of each modality. Unlike transcriptomics, where normalization methods such as scTransform and scran have become well-established (Tran et al., 2020), single-cell proteomics derived from mass spectrometry requires specialized preprocessing to address unique challenges, including missing value imputation, intensity normalization between peptides with differing ionization efficiencies, and variation of cell size-dependent protein content (Brunner et al., 2022; Guo et al., 2025). These modality-specific preprocessing requirements introduce asymmetry in data quality and feature coverage that must be explicitly addressed during integration. Furthermore, the choice of normalization strategy can substantially impact downstream network inference, as methods that over-correct for technical variation may inadvertently suppress biologically meaningful regulatory relationships between transcripts and proteins (Tran et al., 2020; Brunner et al., 2022).

Batch Effect Correction in Unpaired Settings

Batch effects pose a particularly complex challenge in unpaired multi-omics integration (Cao and Gao, 2022). Although methods such as Harmony and BBKNN have demonstrated the efficacy of batch correction within the modality in transcriptomics (Korsunsky et al., 2019), unpaired integration presents a fundamentally more difficult problem. The absence of paired measurements precludes direct alignment of corresponding cells (Cao and Gao, 2022). Recent deep learning frameworks have begun to address this challenge through several strategies: incorporating batch as a decoder covariate to explicitly model batch-dependent variation (Cao and Gao, 2022), employing adversarial training to learn batch-invariant representations (Xu et al., 2024), or using hierarchical variational autoencoders that separate batch effects from biological signals in the latent space (Sun et al., 2025). However, the field still lacks consensus on optimal batch correction strategies that preserve subtle biological variation critical for network inference applications.

Scalability to Atlas-Level Datasets

A critical bottleneck arises when scaling unpaired integration methods to atlas-level datasets that exceed one million cells. Traditional graph-based methods such as GLUE rely on the computing of global pairwise coupling matrices between cells in all modalities, resulting in quadratic computational complexity that renders them impractical for large-scale applications (Sun et al., 2025; Cao and Gao, 2022). Transformer-based architectures such as scmFormer leverage efficient attention mechanisms and mini-batch training strategies, successfully integrating 1.48 million cells from COVID-19 datasets on standard laptop hardware (Xu et al., 2024). Similarly, generative frameworks such as scMRDR avoid explicit pairwise computations by learning latent distributions that can be efficiently sampled and decoded, maintaining flexibility to integrate multiple omics layers without incurring prohibitive memory requirements (Sun et al., 2025). The interpretability of learned representations, a crucial consideration for network inference, may degrade as the complexity of the model increases to accommodate larger datasets (Xu et al., 2024). As single-cell atlases continue to grow in size and complexity, the development of methods that balance computational efficiency, biological fidelity, and interpretability remains an active area of research.

Summary of Methods

Table 1 provides a structured overview of the computational methods reviewed in the main text, organised by their primary function: network inference or network integration and analysis. For each method we list the algorithmic subcategory, the data modalities on which it operates, and the corresponding reference. Methods are grouped to reflect the categorical discussion in the main text, and the table is intended as a quick-reference companion to that discussion.

Table 1 Summary of Methods

Category	Advantage / Disadvantage	Method	Subcategory	Data	Ref.
**Network Inference**					
Knowledge Graph-Based	Validated, simple / Static, limited to prior knowledge.	PrimeKG	Multiple KG	Proteins, diseases, pathways, drugs etc.	(Chandak et al., 2023)
Probabilistic Graphical Models	Direct interactions, Sparse / Data-dependent.	AhGlasso	GGM	Protein	(Zhuang et al., 2022)
		piMGM	MGM	RNA, Cancer Subtype, CNV	(Manatakis et al., 2018)
		BDMCMC	GGM, Bayesian	Gene	(Mohammadi and Wit, 2015)
Boolean	Dynamic, Executable / Overly Simplistic.	PLBIN	Boolean	Protein, RNA, scRNA	(Ye and Guo, 2022)
		mBONITA	Boolean	Protein, Phosphoprotein	(Palshikar et al., 2023)
Generative & Foundation	Causality, Generalizability / Comp. Expensive.	DigNet	Diffusion	scRNA-seq	(Wang and Liu, 2025)
		Planet	Diffusion (Attn)	scRNA-seq	(Xu et al., 2025)
		scRegNet	Hybrid (FM+GNN)	scRNA-seq, TF binding	(Kommu et al., 2025)
**Network Integration and Analysis**					
Matrix Factorization	Straightforward, interpretable / Limited to linear transformations.	MDN-NMTF	NMF (Tri-factorization)	miRNA, Disease descriptors	(Peng et al., 2021)
		UINMF	NMF	scRNA, scATAC, Targeted Spatial RNA	(Kriebel and Welch, 2022)
Network Propagation	Long-range interactions / Topology-dependent and expensive	BRWRMHMDA	Biased RWR	miRNA, Disease descriptors	(Qu et al., 2021)
		unnamed	RWR, HD	RNA, Protein	(Cowen et al., 2017)
		HotNet2	HD	Gene	(Leiserson et al., 2015)
Graph Neural Networks	Flexible, applicable to large datasets / Limited interpretability.	DeepMAPS	HGT	scRNA, scATAC, CITE-Seq	(Ma et al., 2021)
		GLUE	GVAE	scRNA, scATAC, snmC	(Cao et al., 2021)
		scMRDR	Disentangled VAE	scRNA, scMS	(Sun et al., 2025)
		scTGCN	GCN	scRNA, scMS	(Kan et al., 2025)
Consensus	Robustness / Method dependency	COFFEE	Ensemble	scRNA, scATAC	(K Lodi et al., 2024)
		Shusi	LLM + PPI	scRNA, PPI	(Zhang et al., 2025)

Getting Started with Single-Cell Network-Based Multiomics

For researchers new to single-cell network-based multiomics modelling, Table 2 below provides a curated collection of publicly available datasets and computational tools to facilitate entry into the field. Several high-quality CITE-seq and ASAP-seq datasets are freely accessible, offering paired measurements of transcriptomes with surface proteins or chromatin accessibility, respectively (Mimitou et al., 2021; Hao et al., 2021). The 10X Genomics public dataset repository provides standardised preprocessing pipelines and demo data for Multiome (paired RNA+ATAC) experiments, enabling reproducible analysis workflows. For unpaired multiomics integration, the central challenge addressed in this review, we recommend starting with scGLUE (Cao and Gao, 2022), which explicitly models regulatory relationships through prior knowledge graphs and has demonstrated robust performance across diverse tissue types. Researchers working with atlas-scale datasets that exceed hundreds of thousands of cells should consider scJoint (Lin et al., 2022) or scMRDR (Sun et al., 2025), both of which employ efficient training strategies that scale to millions of cells. For paired CITE-seq data, totalVI (Gayoso et al., 2021) provides a probabilistic framework implemented within the scvi-tools ecosystem, offering seamless integration with standard single-cell analysis workflows in Python. Finally, the SCMMIB benchmark framework (Fu et al., 2025) provides standardized evaluation metrics and preprocessing

Table 2 Resources for Single-Cell Network-Based Multiomics Analysis

Resource Type	Name	Description/Link
Public Datasets		
CITE-seq	10X Genomics PBMC	8,617 cord blood mononuclear cells with 228 proteins (Hao et al., 2021)<br>satijalab@GitHub
ASAP-seq	Human bone marrow	Chromatin accessibility + 242 proteins + mtDNA (Mimitou et al., 2021)<br>GEO: GSE156478
Multiome	10X Genomics Demo	Paired scRNA-seq + scATAC-seq<br>10xgenomics website
CITE-seq Atlas	Immune Cell Atlas	more than 200,000 cells with surface markers (Hao et al., 2021)<br>Satijalab website
Integration Methods & Codebases		
Unpaired Integration	scGLUE	Graph-linked embedding with regulatory network inference<br>Gao-lab@GitHub (Cao and Gao, 2022)
	scJoint	Transfer learning for atlas-scale RNA+ATAC<br>SydneyBioX@GitHub (Lin et al., 2022)
	scMRDR	Scalable framework for 1M+ cells<br>PeterZZQ@GitHub (Sun et al., 2025)
Paired Integration	totalVI	Joint analysis of CITE-seq (RNA+protein)<br>scvi-tools website (Gayoso et al., 2021)
	Mowgli	Multi-omics integration with optimal transport<br>cantinilab@GitHub (Huizing et al., 2023)
	MultiMAP	Manifold alignment for batch correction<br>Teichlab@GitHub (Jain et al., 2021)
Benchmarking & Evaluation		
Benchmark	SCMMIB	Systematic evaluation framework for integration methods<br>bm2-lab@GitHub (Fu et al., 2025)
Portal	ASAP	Automated single-cell analysis portal with curated datasets<br>ASAP website

pipelines, enabling objective comparison of integration methods and facilitating method selection based on specific research questions.

References

N. A. Adossa, S. Khan, K. T. Rytkönen, and L. L. Elo. Computational strategies for single-cell multi-omics integration. *Computational and structural biotechnology journal*, 19:2588–2596, Jan. 2021. doi: 10.1016/j.csbj.2021.04.060.

R. Argelaguet, A. S. E. Cuomo, O. Stegle, and J. C. Marioni. Computational principles and challenges in single-cell data integration. *Nature Biotechnology*, 39(10):1202–1215, Oct. 2021. doi: 10.1038/s41587-021-00895-7.

A.-D. Brunner, M. Thielert, C. Vasilopoulou, C. Ammar, F. Coscia, A. Mund, O. B. Hoerning, N. Bache, A. Apalategui, M. Lubeck, et al. Ultra-high sensitivity mass spectrometry quantifies single-cell proteome changes upon perturbation. *Molecular systems biology*, 18(3):e10798, 2022. doi: 10.15252/msb.202110798.

Z.-J. Cao and G. Gao. Multi-omics single-cell data integration and regulatory inference with graph-linked embedding. *Nature Biotechnology*, 40(10):1458–1466, 2022. doi: 10.1038/s41587-022-01284-4.

Z.-J. Cao, Z. Cao, and G. Gao. Multi-omics integration and regulatory inference for unpaired single-cell data with a graph-linked unified embedding framework. *bioRxiv*, Aug. 2021. doi: 10.1101/2021.08.22.457275.

P. Chandak, K. Huang, and M. Zitnik. Building a knowledge graph to enable precision medicine. *Scientific Data*, 10(1):67, Feb. 2023. doi: 10.1038/s41597-023-01960-3.

S. Chen, B. B. Lake, and K. Zhang. High-throughput sequencing of the transcriptome and chromatin accessibility in the same cell. *Nature biotechnology*, 37(12):1452–1457, 2019. doi: 10.1038/s41587-019-0290-0.

L. Cowen, T. Ideker, B. J. Raphael, and R. Sharan. Network propagation: A universal amplifier of genetic associations. *Nature Reviews Genetics*, 18(9):551–562, Sept. 2017. doi: 10.1038/nrg.2017.38.

S. Fu, S. Wang, D. Si, G. Li, Y. Gao, and Q. Liu. Benchmarking single-cell multi-modal data integrations. *Nature Methods*, pages 1–12, 2025. doi: 10.1038/s41592-025-02737-9.

A. Gayoso, Z. Steier, R. Lopez, J. Regier, K. L. Nazor, A. Streets, and N. Yosef. Joint probabilistic modeling of single-cell multi-omic data with totalvi. *Nature methods*, 18(3):272–282, 2021. doi: 10.1038/s41592-020-01050-x.

S. Guo, S. Zhou, G. Wang, and F. Wang. Scpline: An interactive framework for the single-cell proteomics data preprocessing. *Briefings in Bioinformatics*, 26(3), 2025. doi: 10.1093/bib/bbaf256.

Y. Hao, S. Hao, E. Andersen-Nissen, W. M. Mauck, S. Zheng, A. Butler, M. J. Lee, A. J. Wilk, C. Darby, M. Zager, et al. Integrated analysis of multimodal single-cell data. *Cell*, 184(13):3573–3587, 2021. doi: 10.1016/j.cell.2021.04.048.

G.-J. Huizing, I. M. Deutschmann, G. Peyré, and L. Cantini. Paired single-cell multi-omics data integration with mowgli. *Nature Communications*, 14(1):7711, 2023. doi: 10.1038/s41467-023-43019-2.

M. S. Jain, K. Polanski, C. D. Conde, X. Chen, J. Park, L. Mamanova, A. Knights, R. A. Botting, E. Stephenson, M. Haniffa, et al. Multimap: dimensionality reduction and integration of multimodal data. *Genome biology*, 22(1):346, 2021. doi: 10.1186/s13059-021-02565-y.

M. K Lodi, A. Chernikov, and P. Ghosh. COFFEE: consensus single cell-type specific inference for gene regulatory networks. *Briefings in Bioinformatics*, 25(6):bbae457, Sept. 2024. ISSN 1477-4054. doi: 10.1093/bib/bbae457.

Y. Kan, Y. Qi, Z. Zhang, X. Liang, W. Wang, and S. Jin. Integration of unpaired single cell omics data by deep transfer graph convolutional network. *PLOS Computational Biology*, 21(1):e1012625, 2025. doi: 10.1371/journal.pcbi.1012625.

S. Kommu, Y. Wang, Y. Wang, and X. Wang. Prediction of gene regulatory connections with joint single-cell foundation models and graph-based learning. *Bioinformatics*, 41(Supplement_1): i619–i627, July 2025. ISSN 1367-4811. doi: 10.1093/bioinformatics/btaf217.

I. Korsunsky, N. Millard, J. Fan, K. Slowikowski, F. Zhang, K. Wei, Y. Baglaenko, M. Brenner, P.-r. Loh, and S. Raychaudhuri. Fast, sensitive and accurate integration of single-cell data with harmony. *Nature methods*, 16(12): 1289–1296, 2019. doi: 10.1038/s41592-019-0619-0.

A. R. Kriebel and J. D. Welch. UINMF performs mosaic integration of single-cell multi-omic datasets using nonnegative matrix factorization. *Nature Communications*, 13(1):780, Feb. 2022. doi: 10.1038/s41467-022-28431-4.

B. Lee, S. Zhang, A. Poleksic, and L. Xie. Heterogeneous Multi-Layered Network Model for Omics Data Integration and Analysis. *Frontiers in Genetics*, 10:1381, Jan. 2020. doi: 10.3389/fgene.2019.01381.

D. D. Lee and H. S. Seung. Learning the parts of objects by non-negative matrix factorization. *Nature*, 401(6755):788–791, Oct. 1999. doi: 10.1038/44565.

M. D. M. Leiserson, F. Vandin, et al. Pan-cancer network analysis identifies combinations of rare somatic mutations across pathways and protein complexes. *Nature Genetics*, 47(2):106–114, Feb. 2015. doi: 10.1038/ng.3168.

Y. Lin, T.-Y. Wu, S. Wan, J. Y. Yang, W. H. Wong, and Y. R. Wang. scjoint integrates atlas-scale single-cell rna-seq and atac-seq data with transfer learning. *Nature biotechnology*, 40(5): 703–710, 2022. doi: 10.1038/s41587-021-01161-6.

A. Ma, X. Wang, et al. Biological network inference from single-cell multi-omics data using heterogeneous graph transformer. *bioRxiv*, Nov. 2021. doi: 10.1101/2021.10.31.466658.

D. V. Manatakis, V. K. Raghu, and P. V. Benos. piMGM: Incorporating multi-source priors in mixed graphical models for learning disease networks. *Bioinformatics*, 34(17):i848–i856, Sept. 2018. doi: 10.1093/bioinformatics/bty591.

E. P. Mimitou, C. A. Lareau, K. Y. Chen, A. L. Zorzetto-Fernandes, Y. Hao, Y. Takeshima, W. Luo, T.-S. Huang, B. Z. Yeung, E. Papalexi, et al. Scalable, multimodal profiling of chromatin accessibility, gene expression and protein levels in single cells. *Nature biotechnology*, 39(10):1246–1258, 2021. doi: 10.1038/s41587-021-00927-2.

A. Mohammadi and E. C. Wit. Bayesian Structure Learning in Sparse Gaussian Graphical Models. *Bayesian Analysis*, 10(1), Mar. 2015. doi: 10.1214/14-BA889.

M. G. Palshikar, X. Min, A. Crystal, J. Meng, S. P. Hilchey, M. S. Zand, and J. Thakar. Executable Network Models of Integrated Multiomics Data. *Journal of Proteome Research*, 22(5):1546–1556, May 2023. ISSN 1535-3893. doi: 10.1021/acs.jproteome.2c00730. Publisher: American Chemical Society.

W. Peng, J. Du, W. Dai, and W. Lan. Predicting miRNA-Disease Association Based on Modularity Preserving Heterogeneous Network Embedding. *Frontiers in Cell and Developmental Biology*, 9, 2021. doi: 10.3389/fcell.2021.603758.

J. Qu, C.-C. Wang, et al. Biased Random Walk With Restart on Multilayer Heterogeneous Networks for MiRNA–Disease Association Prediction. *Frontiers in Genetics*, 12:720327, Aug. 2021. doi: 10.3389/fgene.2021.720327.

M. Stoeckius, C. Hafemeister, W. Stephenson, B. Houck-Loomis, P. Chattopadhyay, and H. Swerdlow. Simultaneous epitope and transcriptome measurement in single cells. *Nature methods*, 14(9):865–8, 2017. doi: 10.1038/nmeth.4380.

T. Stuart, A. Butler, P. Hoffman, C. Hafemeister, E. Papalexi, W. M. Mauck, Y. Hao, M. Stoeckius, P. Smibert, and R. Satija. Comprehensive Integration of Single-Cell Data. *Cell*, 177(7): 1888–1902.e21, June 2019. ISSN 1097-4172. doi: 10.1016/j.cell.2019.05.031.

J. Sun, C. Liang, R. Wei, P. Zheng, L. Bai, W. Ouyang, H. Yan, and P. Ye. scmrdr: A scalable and flexible framework for unpaired single-cell multi-omics data integration. *arXiv preprint arXiv:2510.24987*, 2025. doi: 10.48550/arXiv.2510.24987.

H. T. N. Tran, K. S. Ang, M. Chevrier, X. Zhang, N. Y. S. Lee, M. Goh, and J. Chen. A benchmark of batch-effect correction methods for single-cell rna sequencing data. *Genome biology*,

21(1):12, 2020. doi: 10.1186/s13059-019-1850-9.

C. Wang and Z.-P. Liu. Diffusion-based generation of gene regulatory networks from scrna-seq data with dignet. *Genome Research*, 35(2):340–354, 2025. doi: 10.1101/gr.279551.124.

J. Xu, D.-S. Huang, and X. Zhang. scmformer integrates large-scale single-cell proteomics and transcriptomics data by multi-task transformer. *Advanced Science*, 11(19):2307835, 2024. doi: 10.1002/advs.202307835.

S. Xu, N. Yu, D. Zhang, and C. Wang. Attention-Guided Probabilistic Diffusion Model for Generating Cell-Type-Specific Gene Regulatory Networks from Gene Expression Profiles. *Genes*, 16(11):1255, Nov. 2025. ISSN 2073-4425. doi: 10.3390/genes16111255. Publisher: Multidisciplinary Digital Publishing Institute.

Q. Ye and N. L. Guo. Inferencing Bulk Tumor and Single-Cell Multi-Omics Regulatory Networks for Discovery of Biomarkers and Therapeutic Targets. *Cells*, 12(1):101, Dec. 2022. doi: 10.3390/cells12010101.

T. Zhang, J. Yu, S. Lou, Y. Liang, Y. Liang, Z. Li, H. Wang, S. Pei, and N. Shen. Systematic discovery of single-cell protein networks in cancer with Shusi, Nov. 2025. ISSN: 2692-8205 Pages: 2025.04.27.649905 Section: New Results.

Y. Zhuang, F. Xing, et al. An Augmented High-Dimensional Graphical Lasso Method to Incorporate Prior Biological Knowledge for Global Network Learning. *Frontiers in Genetics*, 12:760299, Jan. 2022. doi: 10.3389/fgene.2021.760299.
